# Characterization, Comparison of Two New Mitogenomes of Crocodile Newts *Tylototriton* (Caudata: Salamandridae), and Phylogenetic Implications

**DOI:** 10.3390/genes13101878

**Published:** 2022-10-17

**Authors:** Jin-Xiu Wang, Xiang-Ying Lan, Qing-Hua Luo, Zhi-Rong Gu, Qiang Zhou, Ming-Yao Zhang, You-Xiang Zhang, Wan-Sheng Jiang

**Affiliations:** 1 Hunan Engineering Laboratory for Chinese Giant Salamander’s Resource Protection and Comprehensive Utilization, Key Laboratory of Hunan Forest Products and Chemical Industry Engineering, Jishou University, Zhangjiajie 427000, China; 2 College of Biology and Environmental Sciences, Jishou University, Jishou 416000, China; 3 National Nature Reserve of Badagongshan, Zhangjiajie 427100, China

**Keywords:** mitochondrial genome, mitogenome, phylogenetic relationships, *Tylototriton broadoridgus*, *Tylototriton gaowangjienensis*

## Abstract

Mitochondrial genomes (mitogenomes) are valuable resources in molecular and evolutionary studies, such as phylogeny and population genetics. The complete mitogenomes of two crocodile newts, *Tylototriton broadoridgus* and *Tylototriton gaowangjienensis*, were sequenced, assembled, and annotated for the first time using next-generation sequencing. The complete mitogenomes of *T. broadoridgus* and *T. gaowangjienensis* were 16,265 bp and 16,259 bp in lengths, which both composed of 13 protein-coding genes (PCGs), 2 rRNA genes, 22 tRNA genes, and 1 control region. The two mitogenomes had high A + T content with positive AT-skew and negative GC-skew patterns. The ratio of non-synonymous and synonymous substitutions showed that, relatively, the *ATP8* gene evolved the fastest and *COI* evolved the slowest among the 13 PCGs. Phylogenetic trees from BI and ML analyses resulted in identical topologies, where the *Tylototriton* split into two groups corresponding to two subgenera. Both *T. broadoridgus* and *T. gaowangjienensis* sequenced here belonged to the subgenus *Yaotriton*, and these two species shared a tentative sister group relationship. The two mitogenomes reported in this study provided valuable data for future molecular and evolutionary studies of the genus *Tylotoriton* and other salamanders.

## 1. Introduction

Vertebrate mitochondrial genome (mitogenome) is double-stranded circular DNA, typically 16–17 kb in length [1,2]. It encodes, usually, 13 protein-coding genes (PCGs), 2 rRNA genes, 22 tRNA genes, and 1 non-coding control region (CR) that contains information for initiating and regulating gene replication and transcription [3,4,5]. The mitogenome has many characteristics, such as low levels of recombination, multiple copy numbers, simple structure with conserved coding regions, rapid evolutionary rate, and maternal inheritance [6]. For some of these features, mitochondrial DNAs (mtDNAs) have been extensively used as molecular markers for reconstructing phylogenetic relationships, revealing population genetic structures, estimating divergence times, identifying relatedness between recently diverged species, etc. [7,8].

The salamandrid genus *Tylototriton* mainly inhabit montane waterside areas throughout the eastern Himalaya to Indo-China peninsular, including India, Nepal, Myanmar, Thailand, Laos, Vietnam, and central and southern China [9]. The species of this genus are known as crocodile newts because they have a very peculiar appearance like crocodiles: a flat head, large mouth, and highly rough skin with varying-sized warts lined on the dorsal surface. Most of the body surface is pitch black, and some species have prominently red warts or a red tail that looks very striking and flaming. In a phylogenetic view, *Tylototriton* has been known as a group of primitive newts with a sister group relationship to *Echinotriton* [10,11,12]. Although the classification of *Tylototriton* was once debated for being subdivided into different groups [13,14], current studies have widely recognized two subgenera as *Tylototriton* and *Yaotriton* [15], also known as the *T. verrucosus* group and *T. asperrimus* group, respectively [16].

In morphology, species in the subgenus *Tylototriton* usually have orange spots on the body and tail regions, especially prominent on the cranial and dorsal ridges; on the contrary, few orange spots can be seen in the subgenus *Yaotriton* [17]. However, these morphological features might be useful to roughly distinguish the subgenus, but not completely exclusive; thus, the taxonomic studies of the species described recently have been usually carried out based on both morphological and molecular evidence [18,19]. By far, the molecular phylogenetic relationships have showed that *Tylototriton* could divide into two major clades, widely consistent with the classification of the two subgenera suggested by the morphology [7,14,18]. The subgenus *Yaotriton*, additionally, can be divided into two subgroups. Group I is characterized by the formation of dense fistulas on the lateral trunk in a continuous longitudinal row, with thinner transverse veins between the fistulas and a lack of obvious spacing, including species such as *T. wenxianensis*, *T. dabienicus*, *T. broadoridgus*, and *T. liuyangensis*. Group II is characterized by having large nodular fistulas on the dorsolateral side, and with a clear boundary between the fistulas, which consist of *T. asperrimus*, *T. hainanensis*, *T. vietnamensis*, *T. notialis*, and *T. lizhenchangi*. Although the two groups may still have some unidentified new species, as the recent phylogenetic trees revealed [7,18,20], these two subgroups of *Yaotriton*, interestingly, can be divided through clear geographical boundaries: group I is mainly distributed in central China, and group II is distributed in southern China and adjacent areas [17].

*T. broadoridgus* is a species belonging to group I of subgenus *Yaotriton* according to both morphological and molecular studies [7,18,20]. It is a threatened species and has been listed in the second class of the National Key Protected Wild Animal of China [21], with known distribution areas only including Wufeng County in Hubei Province, and Sangzhi County and Liuyang City in Hunan Province [22]. Following field surveys in recent years, we have collected a few individuals of *Tylototriton* in the type locality of *T. broadoridgus* (Sangzhi County) and another place out of its originally known distribution, namely, Guzhang County in Hunan Province. The *Tylototriton* collected from Sangzhi County was identified as *T. broadoridgus* for having the most striking diagnostic characteristics: broad and thick dorsal ridges, with width approximately equal to eye diameter; tail height greater than width at base of tail [22]. However, the *Tylototriton* collected from Guzhang County has relatively narrow (vs. broad) dorsal ridges, and with a separated (vs. connected) tip of “∧” shaped vomerine teeth that distinguished it from *T. broadoridgus*. According to our comprehensively morphological and molecular studies, the *Tylototriton* from Guzhang County were a newly identified species, which we have named as *T. gaowangjienensis* and described in another paper [23].

Both *T. broadoridgus* and *T. gaowangjienensis* are small newts that live in forests with relatively high densities in small creeks or ponds that are required during breeding seasons. Here in this study, we report the complete mitogenomes of both *T. broadoridgus* and *T. gaowangjienensis* that are based on one individual from each of the type localities. As far as we know, the mitogenomes of the two species here are reported for the first time, and we believe these data will be helpful to the studies of population genetics, phylogenetic relationships, and conservation biology of the two rare species, as well as to other *Tylototriton* salamanders in the future.

## 2. Materials and Methods

### 2.1. Sample Collection and Sequencing

Samples of *T. broadoridgus* were collected from the type locality, Badagongshan National Nature Reserve in Sangzhi County in Hunan Province, China. *T. gaowangjienensis* was collected from Gaowangjie National Nature Reserve in Guzhang County, and also in Hunan Province of China. The permissions of field survey for scientific purposes were approved by the local Bureau of National Nature Reserve, and the collection of newts used in this study complied with the Wildlife Protection Act of China. According to the “3R principle” (Reduction, Replacement, and Refinement) of animal sampling, only one sample in each population was used in this study. All the procedures of animal collection and treatment were complied with the guidance of the Code of Practice for the Housing and Care of Animals. The specimens were brought back into the laboratory smoothly, and then euthanatized and preserved in 95% alcohol as voucher specimens deposited in Jishou University (*T. broadoridgus*, voucher no. JWS20221095; *T. gaowangjienensis*, voucher no. JWS20210100). A small part of the tail samples was used for molecular analysis of the two species. The total DNA was extracted using the DNeasy Blood & Tissue Kit (Qiagen, Hilden, Germany), and then the DNA library was constructed, and high-throughput sequencing was conducted in paired-end mode on the DNBSEQ-T7 platform (Complete Genomics and MGI Tech, Shenzhen, China). As the estimated genome size was 25 Gb in *Tylototriton*, approximately 100 Gb raw reads of each sample, with 150 bp read length, were finally generated.

### 2.2. Sequence Assembly, Annotation, and Analysis

The complete mitogenomes of the two samples were assembled using three popular tools, the NOVOPlasty 4.3 [24], MitoZ [25], and MEANGS [26], to increase the success rate and facilitate the mutual correction of undefined sites. The annotation of the final assembled mitogenomes was conducted within the online servers of both MITOS2 [27] and GeSeq [28]. The tRNAscan-SE 1.21 online tool was adopted to predict the secondary structure of tRNAs [29]. The nucleotide composition and codon usage of PCGs were calculated using MEGA 11.0 [30]. The AT skew and GC skew were analyzed using the formula: AT-skew = [A − T]/[A + T] and GC-skew = [G − C]/[G + C] [1]. The ratio of non-synonymous (Ka) and synonymous (Ks) substitutions were calculated using DNASP 6.0 [31] based on 12 species of *Tylototritons* (10 species were downloaded from NCBI). The plots of codon usage frequencies and Ka/Ks ratio were drawn using the Origin software [32].

### 2.3. Phylogenetic Analysis

To reveal the phylogenetic position of the two species we sequenced in this study, another 31 species of Caudata were downloaded from NCBI, whereas the *Batrachuperus pinchonii* in the family Hynobiidae was selected as the outgroup. All of the 13 PCGs were extracted and checked manually through MEGA 11.0 [30], and then each of the PCG alignments based on 33 species were concatenated to make a combined dataset. The best-fit partitioning scheme and partition-specific models were calculated using Partitionfinder 2.1.1 [33], and the sites of codons 1, 2, and 3 of each PCG were assigned. Phylogenetic relationships were reconstructed under Bayesian inference (BI) and maximum likelihood (ML) methods. BI trees were analyzed using MrBayes 3.2.6 [34], running 1,000,000 generations and sampling every 100 generations; after discarding the first 25% samples as burn-in, posterior probabilities (PP) were calculated into a consensus tree. ML trees were performed using RaxML 8.0.2 [35] by executing 10 runs of random additional sequences and generating the bootstrap values following 1000 rapid bootstrap replicates.

## 3. Results

### 3.1. Mitogenome Assembly and Undefined Sites Identification

NOVOPlasty was capable to assemble the fully circled mitogenomes of both samples, with a length of 16,265 bp for *T. broadoridgus* and 16,259 bp for *T. gaowangjienensis*. However, the MitoZ can only fully assemble the sample of *T. broadoridgus*, and the MEANGS can only assemble several fragments (or contigs) for both species. The results of NOVOPlasty, although better-resolved, revealed two undefined sites (probably SNPs) in *T. gaowangjienensis* that presented as degenerate codons, including the loci of Y (2976) and Y (4110). However, these sites assembled from other two tools were presented either as defined sites (MitoZ) or SNPs within multiple assembled fragments (MEANGS). From a conservative concern, we used the assembly results from NOVOPlasty as the basic sequences, and replaced these undefined sites by the results of MitoZ and MEANGS. The two undefined sites of *T. gaowangjienensis* were, therefore, corrected as C (2976) and C (4110) accordingly. The final mitogenomes of *T. broadoridgus* and *T. gaowangjienensis* without any undefined sites were submitted and used for the following analyses.

### 3.2. Mitogenome Annotation and Nucleotide Composition

**Figure 1:**
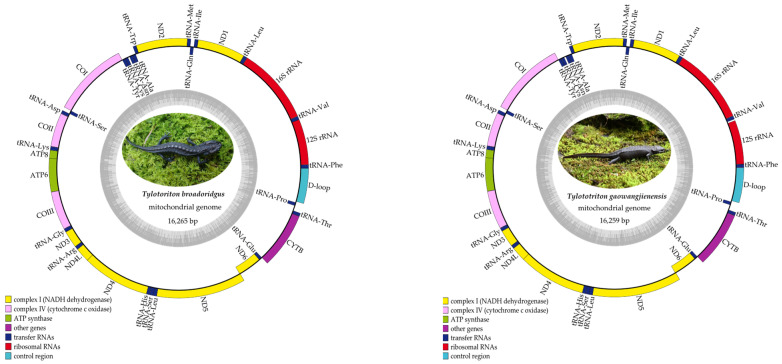
Gene maps of the two newly sequenced *Tylototriton* species.

The complete mitogenomes of *T. broadoridgus* and *T. gaowangjienensis* were 16,265 bp and 16,259 bp in length; of which, both composed 13 PCGs (*ATP6*, *ATP8*, *CYTB*, *ND4L*, *COI-III*, *ND1-6*), 2 rRNA (*12S rRNA* and *16S rRNA*), 22 tRNA genes, and a control region (Figure 1, Table 1). Most of the PCGs, tRNA genes, and rRNA genes were encoded on the heavy strand (H-strand), except *ND6* and eight tRNA genes that encoded on the light strand (L-strand). All of the PCGs of *T. broadoridgus* and *T. gaowangjienensis* started with ATG except the *ATP6* gene in *T. broadoridgus*, and the *COI* gene in both species used GTG as the start codon. There were four kinds of stop codons of the PCGs, whereas the *ND2*, *COI*, *ATP8*, *ATP6*, *ND3*, *NDL*, and *ND5* ended with TAA; the *ND1* ended with TAG; *ND6* ended with AGA; and the *COII*, *COIII*, *ND4*, and *CYTB* used incomplete T(AA) as the stop codon (Table 1). The final mitogenomes of the two species with annotated information have been deposited in GenBank (accession number: OP598114 and ON764431).

Each of the PCGs of the two species was identical in length, but with different lengths in non-PCG regions, such as the *16S* and *12S rRNA* gene in *T. broadoridgus* which were 3 bp and 1 bp longer than that of *T. gaowangjienensis*, respectively (Table 1). Both the mitogenomes of *T. broadoridgus* and *T. gaowangjienensis* contained a total of 41 bp overlapping sites, which were shared in 10 pairs of neighboring genes, ranging from 1 to 15 bp in length. The longest one (15 bp) was overlapped between *ND4L* and *ND4*. For *T. broadoridgus*, a total of 146 bp intergenic nucleotides (IGN) was dispersed in 15 locations, ranging from 1 to 108 bp in length. The longest one (108 bp) was that between *tRNA^Thr^* and *tRNA^Pro^*. The IGN of *T. gaowangjienensis* distributed a similar pattern to that of *T. broadoridgus*, but only had 145 bp sites (Table 1).

The nucleotide compositions of the two species mitogenomes were as follows: (1) *T. broadoridgus*, A (33.6%), T (25.7%), G (14.5%), C (26.3%); and (2) *T. gaowangjienensis*, A (33.6%), T (25.6%), G (14.6%), C (26.3%). The two mitogenomes had similar nucleotide composition patterns, including high A + T contents (59.3% and 59.2%), positive AT-skew (0.13 and 0.14), and negative GC-skew values (both −0.29), which were comparable to other salamander species. For instance, all the 33 salamanders we used for analysis showed positive AT-skews and negative GC-skews, which indicated that the base compositions of salamander mitogenomes were, overall, biased towards A and C (Table 2).

### 3.3. Characteristics of PCGs and Codon Usage

**Figure 2:**
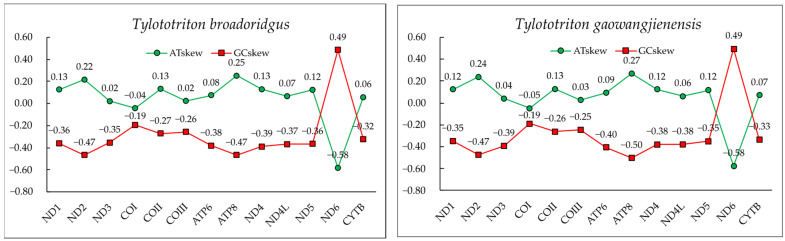
GC and AT skews of mitochondrial PCGs of *T. broadoridgus* and *T. gaowangjienensis*.

The total length of PCGs in both *T. broadoridgus* and *T. gaowangjienensis* were identical to 11,383 bp, with the following base compositions: (1) *T. broadoridgus*, A (31.0%), T (27.3%), G (14.7%), C (27.0%); and (2) *T. gaowangjienensis*, A (31.0%), T (27.2%), G (14.8%), C (27.1%). The mean A + T content values were 58.3% and 58.2% for *T. broadoridgus* and *T. gaowangjienensis*, respectively, with similar positive AT-skew (0.06 and 0.07) and identical negative GC-skew (−0.29) (Table 3). All the PCGs presented positive AT-skews, except a negative value existed in *COI* and *ND6*, and all the PCGs presented negative GC-skews, except a positive value existed in *ND6* (Figure 2). Given that the different codon positions might have different codon bias in PCGs, we also examined nucleotide compositions from the three codon positions of *T. broadoridgus* and *T. gaowangjienensis* (Table 3). Interestingly, the A + T contents were slightly increasing from the first to third codon positions. In detail, there were 55.4% for PCGs-first, 58.7% and 58.4% for PCGs-second, and 60.8% and 60.0% for PCGs-third for the two species, respectively. All of the codons showed positive AT-skews, except the second codon showed a slightly negative value, whereas all GC-skews in three codon positions were negative (Table 3).

**Figure 3:**
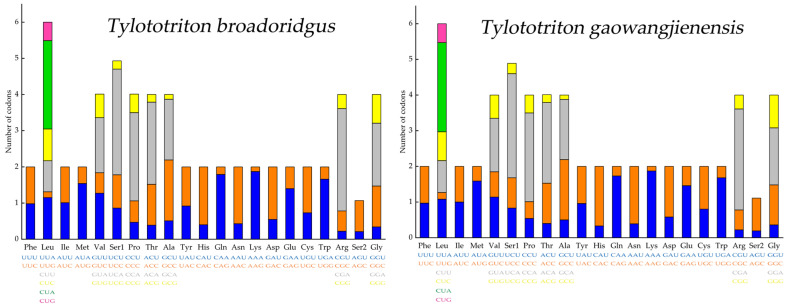
Relative Synonymous Codon Usage (RSCU) of mitogenomes of *T. broadoridgus* and *T. gaowangjienensis*.

**Figure 4:**
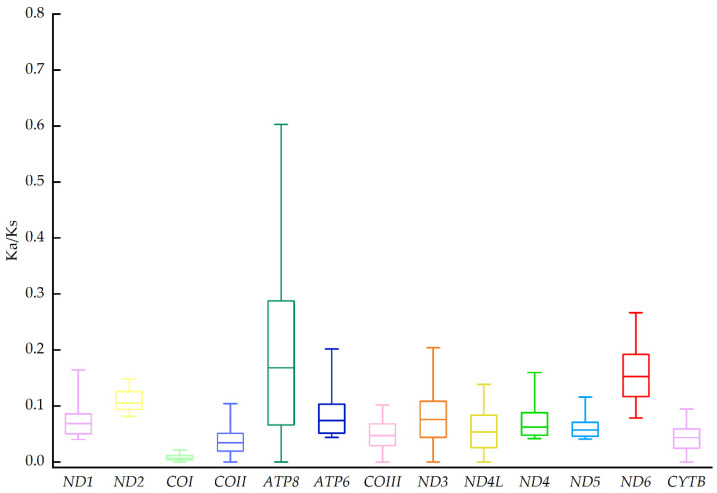
The Ka/Ks ratio of 13 PCGs among 12 species of *Tylototriton*.

Codon usage bias would drive genes to evolve at different rates [36]. Statistics on the relative synonymous codon usage (RSCU) of *T. broadoridgus* and *T. gaowangjienensis* showed they shared very similar patterns (Figure 3). In terms of codon frequencies, the CUA (Leu), CCA (Pro), CGA (Arg), and UCA (Ser1) were the most abundant codons in both *T. broadoridgus* and *T. gaowangjienensis*. The calculation of the Ka/Ks ratio of each PCG would assess the different evolutionary rate [37]. Among the analyzed 12 species of *Tylototriton*, the *ATP8* gene evolved relatively fast and exhibited the highest Ka/Ks value, whereas *COI*, on the contrary, showed the lowest Ka/Ks (Figure 4); however, the Ka/Ks for all 13 PCGs were below 0.7, and did not show positive selection signals.

### 3.4. Characteristics of rRNAs, tRNAs, and the Control Region

There were two rRNA genes of both *T. broadoridgus* and *T. gaowangjienensis*: the *16S rRNA* was located between *tRNA^Val^* and *tRNA^Leu^*, with corresponding lengths of 1563 bp and 1560 bp, whereas the *12S rRNA* was located between *tRNA^Phe^* and *tRNA^Val^*, with corresponding lengths of 928 bp and 927 bp (Table 2). The nucleotide composition of the two rRNAs were similar, and the AT-skews were positive and the GC-skews were negative for the two species (Table 3).

The mitogenomes of both *T. broadoridgus* and *T. gaowangjienensis* contained 22 tRNA genes; of which, eight genes, including *tRNA^Gln^*, *tRNA^Ala^*, *tRNA^Asn^*, *tRNA^Cys^*, *tRNA^Tyr^*, *tRNA^Ser^*, *tRNA^Glu^*, and *tRNA^Pro^*, were on the L-strand, and the rest were on the H-strand (Table 1). The total length of tRNAs was 1537 bp in both species, and the individual length of each tRNA gene was generally identical, except *tRNA^Phe^* and *tRNA^Gly^* have a 1 bp difference between the two species. It ranged from 66–75 bp of all the tRNA genes, with the longest *tRNA^Leu^* and the shortest *tRNA^Cys^* (Table 3). All tRNA genes, except *tRNA^Ser^*, can be folded into a typical cloverleaf structure.

The non-coding control region, also known as the *D-loop*, was usually the sequence region with greatest variations across the mitogenome. Here, the *D-loops* of *T. broadoridgus* and *T. gaowangjienensis* were 716 bp and 715 bp in length, located between *tRNA^Pro^* and *tRNA^Phe^*, with similar A + T contents, AT-skew, and GC-skew values (Table 3).

### 3.5. Phylogenetic Analysis

Phylogenetic trees from BI and ML analyses resulted in almost identical topologies, where the Salamandridae was divided into three clades known as three subfamilies, namely, Salamandrininae, Salamandrinae, and Pleurodelinae. The Salamandrininae contained only one genus, *Salamandrina*, that diverged first, followed by the subfamily, Salamandrinae, well-known as the “True” Salanmanders, which contained genera such as *Lyciasalamandra*, *Salamandra*, *Chioglossa*, and *Mertensiella*. The majority of species were grouped into the subfamily Pleurodelinae, which can be furtherly divided into several well-supported subclades, including the primitive newts (*Echinotriton*, *Pleurodeles*, and *Tylototriton*), New World newts (*Notophthalmus* and *Taricha*), Corsica–Sardinia newts (*Euproctus*), modern Asian newts (*Cynops*, *Paramesotriton*, *Pachytriton*, and *Laotriton*), and modern European newts (*Lissotriton*, *Ichthyosaura*, *Calotriton*, *Triturus*, *Ommatotriton*, and *Neurergus*).

The genus *Tylototriton* was one group of the primitive newts, which divided into, as expected, two major groups corresponding to the two subgenera, *Tylototriton* and *Yaotriton*. The subgenus *Yaotriton* was further divided into two subgroups. The first subgroup included *T. biegleri* and *T. asperrimus*, and the second one included *T. wenxianensis*, and both *T. broadoridgus* and *T. gaowangjienensis* that we sequenced in this study. *T. broadoridgus* and *T. gaowangjienensis* were revealed as sister groups, and then clustered with *T. wenxianensis*. The subgenus *Tylototriton* also divided into two subgroups. The first one included *T. pseudoverrucosus* and *T. taliangensis*, and the second one included five species that diverged in the following sequences: *T. kweichowensis*, *T. shanorum*, *T. yangi*, *T. verrucosus*, and *T. shanjing*.

### 3.6. Species Verification from ND2 and 16S rRNA Gene

The species verification was fundamental for reporting a new mitogenome. Following the suggestions of Sangster and Luksenburg (2021) [38], we verified the identity of our mitogenome sequence of *T. broadoridgus* with reference sequences of two commonly used markers in *Tylotoriton* systematics [7]: the *ND2* (1035 bp; *n* = 107, incl. three of *T. broadoridgus*, KC147814, KY800837, and OK539842) and *16S rRNA* (508 bp; *n* = 89, incl. two of *T. broadoridgus*, KY800569 and KY800570). In each of these analyses, our sequence of *T. broadoridgus* clustered with the reference sequences of *T. broadoridgus*, indicating that our sample was correctly identified. As there are no reference sequences of *T. gaowangjiensis*, we added our newly obtained *ND2* and *16S rRNA* sequences into a previous dataset from a phylogenetic study that included the most species of *Tylotoriton* so far [7]. A simple neighbor-joining tree based on both *ND2* and *16S rRNA* sequences revealed the sister species of *T. gaowangjiensis* was *T. dabienicus*, which was collected from Shangcheng County, Anhui Province of China [7], with genetic distances that ranged from 1% to 2%.

## 4. Discussion

The genome sizes of species would affect the rate of assembly success for both nuclear and organellar genomes, with a putatively positive relationship between genome sizes and sequence complexities [39]. Because the genome sizes of Caudata amphibians were relatively large—for instance, the genome sizes of *Tylotoriton* that we studied were ~25 G [40]—the two samples we sequenced were assembled using three popular tools, NOVOPlasty, MitoZ, and MEANGS, for comparisons. Generally, NOVOPlasty performed best, as it assembled fully circled mitogenomes for both samples; however, it produced several undefined loci that presented as degenerate codons. However, these sites assembled from the other two tools were presented as either defined sites (MitoZ) or SNPs in multiple assembled fragments (MEANGS). This might result from the different strategies of the three software in balancing the assembly performance and error-tolerance rates [24,25,26]. We carried out a strategy to correct the undefined sites from NOVOPlasty with defined sites or more abundant SNPs from MitoZ and MEANGS, and, thus, we obtained the final whole mitogenomes without any undefined sites. This approach would be helpful for the mitogenome assembly of other species with relatively large genome sizes.

As far as we know, the mitogenomes of the two crocodile newts, *T. broadoridgus* and *T. gaowangjienensis*, were assembled in this study for the first time. The characteristics of the mitogenomes of the two species were very similar, in terms of mitogenome size and organization (Figure 1, Table 1); and nucleotide composition of PCGs, rRNAs, tRNAs, control region, or codon usage of PCGs (Figure 2 and Figure 3, Table 3). They also showed very similar patterns with other *Tylototriton* species reported previously [11,41,42,43,44,45,46], even though some minor differences remained. For example, the *ND3* used the incomplete “T--” as the stop codon in *T. wenxianensis* [41], but this gene used the conventional stop codon “TAA” in both *T. broadoridgus* and *T. gaowangjienensis*. Similarly, the *ND5* used “TAG” as the stop codon in *T. taliangensis* [42], but it stopped with “TAA” in both *T. broadoridgus* and *T. gaowangjienensis* (Table 1). Whether the diverse usage of stop codons among the closely-related species was generated randomly or with some meaningful preferences was an interesting question of selection, but was not given much attention.

**Figure 5:**
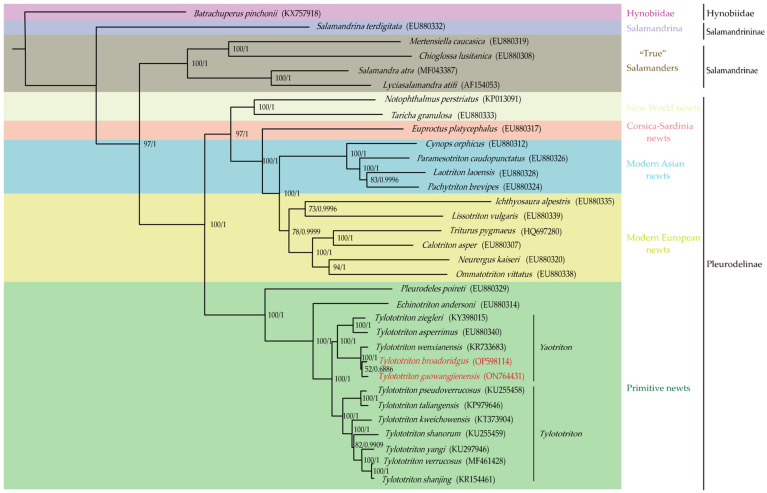
Phylogenetic relationships within Salamandridae derived from ML method based on 13 PCGs. Note: Names in red show the phylogenetic positions of *T. broadoridgus* and *T. gaowangjienensis* that we sequenced in this study. The numbers on the nodes are the bootstrap values and posterior probabilities from ML and BI methods. The GenBank accession number of each species is given in the bracket after the species name.

In this study, the phylogenetic relationships within Salamandridae were able to be reconstructed, while using 32 representative species, including 2 newly obtained in this study, as the ingroups, and *B. pinchonii* in Hynobiidae as the outgroup. The relationship of the three subfamilies of the Salamandridae has been highly supported and broadly consistent with previous studies [11,47]. However, some new, but different, inter-generic relationships were also revealed. For instance, while using only *16S rRNA* and *ND2* as gene markers, Wang et al. (2018) recovered a sister group relationship of *Triturus* and *Neurergus*, but, here, we revealed the sister group of *Triturus* was *Calotriton* (Figure 5). Although the phylogenetic hypotheses would be changed based on different DNA markers [12,48], it was speculated that more sequences used, such as the 13 PCGs here, would be generally better to understand the real phylogenetic relationships.

The crocodile newts, *Tylotoriton*, were recovered as a monophyletic group split into two well-supported subgenera (*Yaotriton* and *Tylotoriton*) (Figure 5). The inter-specific relationships were broadly consistent with the previous findings that were based on several gene fragments [7,14,18,49,50], but some interesting differences were also revealed and are worth attention. For example, Nishikawa et al. (2013) [49] found that in the subgenus *Tylotoriton*, *T. verrucosus* branched off first, followed by *T. shanjing* and *T. yangi*. In contrast, our study showed that *T. yangi* divided first and then grouped with *T. shanjing* plus *T. verrucosus*. Both *T. broadoridgus* and *T. gaowangjienensis* that we sequenced in this study were recovered as members in the subgenus *Yaotriton*, which was consistent with the morphological studies. It was also reasonable in terms of the distribution area of this unique group [17]. Although the phylogenetic tree revealed that *T. broadoridgus* and *T. gaowangjienensis* were sister groups, this relationship would be tentative, as the *Tylotoriton* species with reported mitogenomes were still very limited. The whole picture of the biogeography of *Tylotoriton* would be presented, whereas more molecular data, such as mitogenomes from more species, would be available in the future.

## 5. Conclusions

In summary, we have successfully sequenced and assembled the complete mitogenomes of two rare species of crocodile newts, *T. broadoridgus* and *T. gaowangjienensis*, for the first time. We further provided detailed characteristics of the two mitogenomes in aspects of gene orders, nucleotide composition, and codon usages from different regions, such as rRNAs, tRNAs, PCGs, and the non-coding control region. The phylogenetic trees using the BI and ML methods based on 13 PCGs of 32 species have provided well-supported major clade relationships within Salamandridae for reference, as well as revealed new relationships among *Tylotoriton*, which we were concerned with the most. The two mitogenomes reported here and the detailed analyses in this study would provide valuable materials and data for future taxonomic and evolutionary studies of the genus *Tylotoriton* and other salamanders.

## Figures and Tables

**Table 1:** Mitochondrial genome organization of *T. broadoridgus* (TB) and *T. gaowangjienensis* (TG).

Gene	Position				Length (bp)		Start Codon		Stop Codon		Anticodon	Strand	Intergenic Nucleotide	
	TB		TG		TB	TG	TB	TG	TB	TG				
	From	To	From	To									TB	TG
*tRNA^Phe^*	1	68	1	69	68	69					GAA	H	0	0
*12S rRNA*	69	996	70	996	928	927						H	−1	−1
*tRNA^Val^*	996	1065	996	1065	70	70					TAC	H	2	2
*16S rRNA*	1068	2630	1068	2627	1563	1560						H	1	1
*tRNA^Leu^*	2632	2706	2629	2703	75	75					TAA	H	0	0
*ND1*	2707	3675	2704	3672	969	969	ATG	ATG	TAG	TAG		H	−1	−1
*tRNA^Ile^*	3675	3745	3672	3742	71	71					GAT	H	2	2
*tRNA^Gln^*	3748	3818	3745	3815	71	71					CAT	L	2	1
*tRNA^Met^*	3821	3890	3817	3886	70	70					TCA	H	0	0
*ND2*	3891	4934	3887	4930	1044	1044	ATG	ATG	TAA	TAA		H	−2	−2
*tRNA^Trp^*	4933	5001	4929	4997	69	69					GTC	H	1	1
*tRNA^Ala^*	5003	5071	4999	5067	69	69					TTT	L	0	0
*tRNA^Asn^*	5072	5144	5068	5140	73	73					TCC	L	2	2
*OL*	5147	5179	5143	5175	33	33						H	−1	−1
*tRNA^Cys^*	5179	5244	5175	5240	66	66					TCG	L	0	0
*tRNA^Tyr^*	5245	5311	5241	5307	67	67					GTG	L	1	1
*COI*	5313	6863	5309	6859	1551	1551	GTG	GTG	TAA	TAA		H	0	0
*tRNA^Ser^*	6864	6934	6860	6930	71	71					GCT	L	1	1
*tRNA^Asp^*	6936	7005	6932	7001	70	70					TAG	H	1	1
*COII*	7007	7694	7003	7690	688	688	ATG	ATG	T(AA)	T(AA)		H	0	0
*tRNA^Lys^*	7695	7767	7691	7763	73	73					TGT	H	1	1
*ATP8*	7769	7936	7765	7932	168	168	ATG	ATG	TAA	TAA		H	−10	−10
*ATP6*	7927	8610	7923	8606	684	684	GTG	ATG	TAA	TAA		H	−1	−1
*COIII*	8610	9393	8606	9389	784	784	ATG	ATG	T(AA)	T(AA)		H	0	0
*tRNA^Gly^*	9394	9463	9390	9458	70	69					TGG	H	0	0
*ND3*	9464	9811	9459	9806	348	348	ATG	ATG	TAA	TAA		H	−2	−2
*tRNA^Arg^*	9810	9878	9805	9873	69	69					TTC	H	0	0
*ND4L*	9879	10,175	9874	10,170	297	297	ATG	ATG	TAA	TAA		H	−7	−7
*ND4*	10,169	11,546	10,164	11,541	1378	1378	ATG	ATG	T(AA)	T(AA)		H	0	0
*tRNA^His^*	11,547	11,614	11,542	11,609	68	68					TGA	H	0	0
*tRNA^Ser^*	11,615	11,682	11,610	11,677	68	68					GTA	H	−1	−1
*tRNA^Leu^*	11,682	11,753	11,677	11,748	72	72					GCA	H	0	0
*ND5*	11,754	13,565	11,749	13,560	1812	1812	ATG	ATG	TAA	TAA		H	−15	−15
*ND6*	13,551	14,069	13,546	14,064	519	519	ATG	ATG	AGA	AGA		L	0	0
*tRNA^Glu^*	14,070	14,137	14,065	14,132	68	68					GTT	L	2	2
*CYTB*	14,140	15,280	14,135	15,275	1141	1141	ATG	ATG	T(AA)	T(AA)		H	0	0
*tRNA^Thr^*	15,281	15,348	15,276	15,343	68	68					TGC	H	108	108
*tRNA^Pro^*	15,457	15,527	15,452	15,522	71	71					TTG	L	22	22
*D-loop*	15,550	16,265	15,545	16,259	716	715						H	0	0

**Table 2:** Base composition (in percentages) of the mitogenomes of 33 species in Salamandridae that were used for phylogenetic analyses in this study.

Species	Total Length (bp)	T (%)	C (%)	A (%)	G (%)	A + T Content (%)	AT-Skew	GC-Skew	Accession Number
*T. broadoridgus*	16,265	25.7	26.3	33.6	14.5	59.3	0.13	−0.29	OP598114
*T. gaowangjienensis*	16,259	25.6	26.3	33.6	14.6	59.2	0.14	−0.29	ON764431
*Tylototriton wenxianensis*	16,265	25.67	26.20	33.62	14.51	59.29	0.13	−0.29	KR733683
*Tylototriton kweichowensis*	16,727	25.64	26.10	33.93	14.33	59.57	0.14	−0.29	KT373904
*Tylototriton asperrimus*	16,161	25.51	26.50	33.26	14.73	58.77	0.13	−0.29	EU880340
*Tylototriton pseudoverrucosus*	16,265	26.06	25.77	33.40	14.77	59.46	0.12	−0.27	KU255458
*Tylototriton shanjing*	16,661	25.41	26.28	34.04	14.27	59.45	0.15	−0.30	KR154461
*Tylototriton taliangensis*	16,265	26.03	25.76	33.42	14.79	59.45	0.12	−0.27	KP979646
*Tylototriton verrucosus*	16,660	25.43	26.31	33.99	14.27	59.42	0.14	−0.30	MF461428
*Tylototriton yangi*	16,648	25.53	26.21	33.99	14.28	59.51	0.14	−0.29	KU297946
*Tylototriton ziegleri*	16,266	25.49	26.38	33.71	14.42	59.20	0.14	−0.29	KY398015
*Tylototriton shanorum*	17,096	25.26	26.40	34.11	14.23	59.37	0.15	−0.30	KU255459
*Cynops orphicus*	16,296	28.91	23.16	32.88	15.05	61.79	0.06	−0.21	EU880312
*Echinotriton andersoni*	16,272	26.47	25.44	34.02	14.07	60.49	0.12	−0.29	EU880314
*Euproctus platycephalus*	15,799	30.14	22.09	34.15	13.61	64.30	0.06	−0.24	EU880317
*Calotriton asper*	16,564	26.47	25.93	32.20	15.41	58.66	0.10	−0.25	EU880307
*Ichthyosaura alpestris*	16,339	27.39	25.05	32.91	14.65	60.30	0.09	−0.26	EU880335
*Laotriton laoensis*	16,361	27.30	24.88	31.80	16.03	59.09	0.08	−0.22	EU880328
*Lissotriton vulgaris*	16,310	28.34	24.74	31.76	15.16	60.10	0.06	−0.24	EU880339
*Neurergus kaiseri*	16,202	27.69	23.97	34.57	13.78	62.26	0.11	−0.27	EU880320
*Notophthalmus perstriatus*	16,336	28.34	23.84	34.08	13.74	62.41	0.09	−0.27	KP013091
*Paramesotriton caudopunctatus*	15,968	28.49	23.57	33.73	14.21	62.22	0.08	−0.25	EU880326
*Pleurodeles poireti*	16,211	27.96	24.46	33.26	14.32	61.22	0.09	−0.26	EU880329
*Taricha granulosa*	16,151	24.99	27.67	31.37	15.97	56.36	0.11	−0.27	EU880333
*Lyciasalamandra atifi*	16,650	29.07	24.13	32.34	14.47	61.41	0.05	−0.25	AF154053
*Mertensiella caucasica*	17,023	29.38	24.54	31.96	14.12	61.34	0.04	−0.27	EU880319
*Salamandra atra*	15,592	30.31	23.35	32.45	13.89	62.76	0.03	−0.25	MF043387
*Salamandrina terdigitata*	16,252	29.59	22.26	34.57	13.59	64.16	0.08	−0.24	EU880332
*Chioglossa lusitanica*	16,417	31.03	22.80	33.01	13.16	64.04	0.03	−0.27	EU880308
*Pachytriton brevipes*	16,240	28.18	23.80	33.15	14.87	61.33	0.08	−0.23	EU880324
*Triturus pygmaeus*	16,442	27.25	25.73	31.83	15.19	59.08	0.08	−0.26	HQ697280
*Ommatotriton vittatus*	16,193	28.80	23.84	32.38	14.98	61.18	0.06	−0.23	EU880338
*B. pinchonii*	16,381	32.84	19.65	33.92	13.60	66.75	0.02	−0.18	KX757918

**Table 3:** Nucleotide composition and skewness of the mitogenomes of *T. broadoridgus* (TB) and *T. gaowangjienensis* (TG).

	Size		A (%)		T (%)		C (%)		G (%)		A + T (%)		AT-Skew		GC-Skew	
	TB	TG	TB	TG	TB	TG	TB	TG	TB	TG	TB	TG	TB	TG	TB	TG
*D-loop*	716	715	28.6	29.0	34.9	34.5	21.2	21.0	15.2	15.5	63.5	63.5	−0.10	−0.09	−0.17	−0.15
*12SrRNA*	928	927	37.9	38.1	19.9	20.1	23.9	23.7	18.2	18.1	57.8	58.2	0.31	0.31	−0.14	−0.13
*16SrRNA*	1563	1560	40.2	40.1	23.3	23.4	20.3	20.1	16.3	16.4	63.5	63.5	0.27	0.26	−0.11	−0.10
tRNAs	1537	1537	32.3	32.1	30.1	30.0	17.7	17.9	19.8	20.0	62.4	62.1	0.04	0.03	0.06	0.05
PCGs-1st	3795	3795	30	30.3	25.4	25.1	25.1	25.2	19.4	19.3	55.4	55.4	0.08	0.09	−0.13	−0.13
PCGs-2nd	3794	3794	25.1	25	33.6	33.4	27.9	28	13.5	13.5	58.7	58.4	−0.14	−0.14	−0.35	−0.35
PCGs-3rd	3794	3794	37.8	37	23	23	28.1	28	11.1	11.4	60.8	60.0	0.24	0.23	−0.43	−0.42
PCGs	11,383	11,383	31	31	27.3	27.2	27	27.1	14.7	14.8	58.3	58.2	0.06	0.07	−0.29	−0.29
Genome	16,265	16,259	33.6	33.6	25.7	25.6	26.3	26.3	14.5	14.6	59.3	59.2	0.13	0.14	−0.29	−0.29

## Data Availability

The assembled mitogenome sequences have been deposited in NCBI (https://www.ncbi.nlm.nih.gov/ (accessed on 15 June 2022) with accession number: OP598114 and ON764431. All data generated by this study are available from the corresponding author upon reasonable request.
